# The oncogenic potential of salivary microRNA-93 and microRNA-412-3p in oral lichen planus: a case-control study

**DOI:** 10.1038/s41405-024-00278-5

**Published:** 2024-12-23

**Authors:** Moataz M. ELHefny, Inas A. Korien, Weam AM Rashwan, Olfat G. Shaker

**Affiliations:** 1 Department of Oral Medicine, Oral Diagnosis and Periodontology, Faculty of Dentistry, Horus University, New Damietta, Egypt; 2 Department of Oral Medicine, Oral Diagnosis and Periodontology, Faculty of Dentistry, Cairo University, Cairo, Egypt; 3 Kasr El-Aini Center of Clinical Oncology and Nuclear Medicine, Cairo University, Cairo, Egypt; 4 Oral Medicine, Oral Diagnosis and Periodontology, Department of Oral Medicine, Oral Diagnosis and Periodontology, Faculty of Dentistry, Cairo University, Cairo, Egypt; 5 Medical Biochemistry and Molecular Biology, Faculty of Medicine, Cairo University, Cairo, Egypt

**Keywords:** Oral diseases, Oral cancer detection

## Abstract

**Background:**

Oral Lichen Planus is one of the most popular chronic mucocutaneous diseases. It is classified as potentially malignant lesions. Many microRNAs can be used as biological markers for the disease and for its malignant transformation. The aim of the study to measure the expression of microRNA-93 and microRNA-412-3p in Oral Lichen Planus patients, patients diagnosed as Oral Squamous Cell Carcinoma and healthy controls.

**Methodology:**

A total of 60 patients were divided into 3 groups; each group contains 20 patients. Group I for Oral Lichen Planus patients, group II for healthy controls and group III for Oral Squamous cell carcinoma patients. All of these patients were chosen from those attending Kasr Al-Einy hospital, Cairo University. After full diagnosis and matching of our eligibility criteria, saliva sample was taken from each patient to measure the concentration of microRNA-93 and microRNA-412-3p.

**Results:**

Both microRNA-93 and miceoRNA-412-3p were upregulated in Oral Squamous cell carcinoma patients than Oral Lichen patients than controls and both of them had great sensitivity, specificity and diagnostic accuracy for both Oral Lichen Planus and Oral Squamous cell carcinoma.

**Conclusion:**

MicroRNA-93 and micriRNA-412-3p can be used as diagnostic markers and for the oncogenic potential of Oral Lichen Planus.

## Introduction

Oral lichen planus (OLP) is one of the most common prevalent mucocutaneous chronic diseases. The disease is auto-immune type, and despite having idiopathic etiology, many risk factors can be considered including systemic diseases, psychogenic diseases, dental restorations, and some drugs [1]. The oral lesions are mainly bilateral, frequently appearing in the inner buccal mucosa [2]. OLP has multiple clinical forms in the oral cavity [2]. The disease is a premalignant lesion with rare potential for malignant transformation [3]. T-lymphocyte infiltration in the basal cell layer of the epithelium and cytoid bodies are characteristic histopathologic features of the disease [2]. Genetic malformations and epigenetic mechanisms as microRNAs may have a role in these changes [4].

The first WHO criteria for diagnosis of OLP was published in 1978 and applied for many years [5]. However, these criteria have shown lack of clinicopathologic correlation in the diagnosis of OLP and how to differentiate between OLP and Oral Lichenoid lesion (OLL). So, modified WHO criteria were published in 2003 to define OLP and OLL, including clinical as well as histopathologic criteria, to allow proper diagnosis based on a reproducible manner [6]. The modified criteria strict both clinical and histopathological criteria for definite diagnosis of both lesions. Dysplastic lesions are excluded from the modified criteria.

MicroRNAs (miRNAs) are endogenic short non-coding about 22 nucleotides RNAs in length [7, 8]. MicroRNAs perform major regulatory roles in animals and plants by targeting mRNAs then cleavage or translational repression of mRNAs occurs. MiRNAs belong to one group of molecules that regulate genes, and they affect the protein production of numerous genes [7, 9]. MicroRNAs play a critical role in the development of various diseases in broad pathological conditions [10]. MiRNAs might contribute to biomarkers for risk of development, prognosis, and response to treatment of oral cancer as miR-21, miR-24, miR-134, and miR-146a [11, 12].

MiRNA-93 is a type of miRNA within the miR-106b∼25 cluster and plays a role in promoting cell survival, supporting the formation of spheres, enhancing tumor growth, increasing angiogenesis through endothelial cell activation, and preventing apoptosis by targeting integrin-β8, a protein-associated with cell death [13]. MiRNA-93 expression upregulation was elevated in neuroblastoma, non-small cell lung cancer, breast cancer, and ovarian cancer [14]. Furthermore, it has been detected a significant increase in the expression of miRNA-93 expressed in the saliva of Oral Squamous Cell Carcinoma (OSCC) patients [15].

MiRNA-412-3p has shown promise in predicting cancer-specific mortality and has significant implications for cancer progression [16]. Additionally, it is highly expressed in extracellular vesicles of patients with oral squamous cell carcinoma (OSCC) [17–19].

This study aims to evaluate the oncogenic potential of miRNA-93 and miRNA-412-3p in oral lichen planus patients, providing valuable insights into potential new oncogenic markers for this condition.

## Materials and methods

**Fig. 1:**
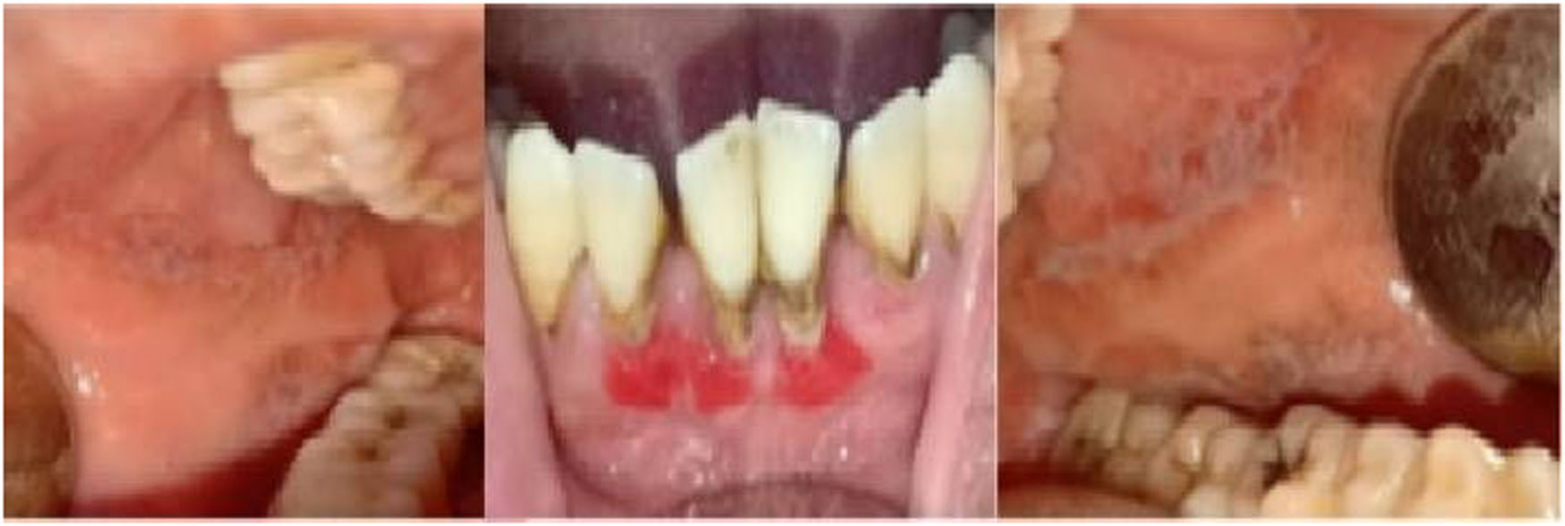
Case No. 1. 33 years old male patient suffering from Oral Lichen Planus in the buccal mucosa bilaterally and desquamative gingivitis.

**Fig. 2:**
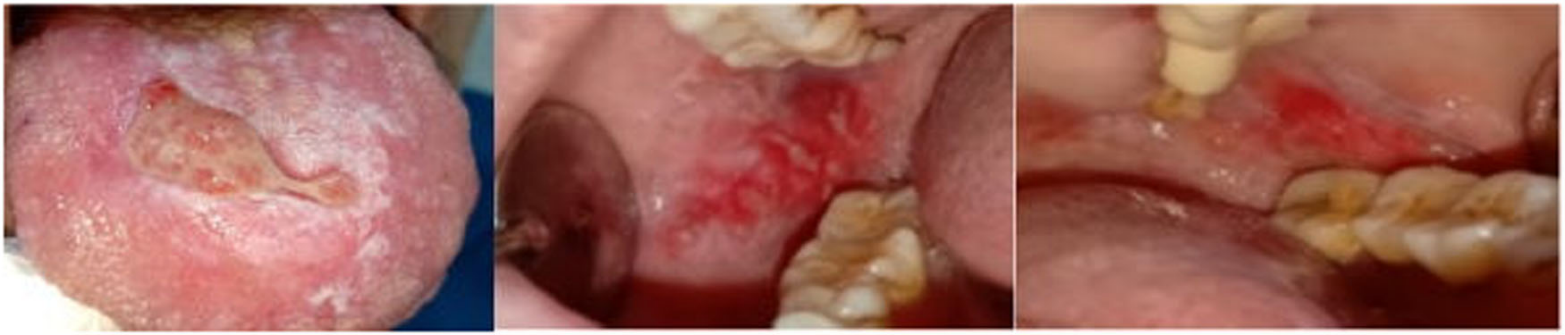
Case No. 2. 59 years old female patient suffering from Oral lichen planus at the tongue and buccal mucosa bilaterally.

The present investigation is an observational case-control study that included 60 patients divided into 3 groups: Group I included 20 patients systemically and orally free except for oral lichen planus lesions, with or without skin or other Lichen manifestations for at least 6 months. These patients were systemically free and not taking any medication in the last three months. Group I patients were selected from the outpatient clinic of the Department of Oral Medicine, Periodontology and Oral Diagnosis, Faculty of Dentistry, Cairo University. The patients in group I were fully examined and diagnosed clinically and histopathologically according to the modified WHO diagnostic criteria of OLP [6]. (Figs. 1 and 2) The patients of this group were chosen according to strict inclusion and exclusion criteria. The inclusion criteria include both genders ranging from 20 to 70 years old, patients who were able to return for follow-up visits, patients who were diagnosed clinically and histopathologically as having symptomatic OLP, and Patients who agreed to write consent after understanding the nature of the study. Exclusion criteria included patients who have systemic or other mucosal lesions, patients taking any topical or systemic medication last three months, pregnant or lactative female patients, and patients who have any degree of dysplasia according to histopathological reports. Group II included 20 healthy control subjects, free from oral lesions, and were chosen from the outpatients attending Dental clinics of the other departments of the Faculty of Dentistry, Cairo University. Group III included 20 OSCC patients selected from the outpatient clinic of the Department of Oncology and Nuclear Medicine, Faculty of Medicine, Cairo University, and diagnosed with oral squamous cell carcinoma before the application of any therapeutic intervention. Exclusion criteria for all groups included any patient with any systemic disease or having medication, vulnerable patients, pregnant, lactating females, smokers, or alcoholic patients. Each patient was informed of the clinical steps of the study and signed a written informed consent for ethical purposes.

Sample size calculation was done using the comparison of salivary miRNA-93 expression between oral lichen planus (OLP), and healthy controls. We depended on the available results on a similar miRNA (miRNA27b) in OLP and healthy controls, as reported in a previous publication (Aghbari et al.) [20], the mean ± SD of miRNA-27b expression in the OLP group was approximately 5.343 ± 4.88, while in the control group, it was approximately 10.592 ± 2.142. Accordingly, we calculated that the minimum proper sample size was 10 participants in each group to be able to detect a real difference of 2 units in miRNA expression with 80% power at α = 0.05 level using Student’s t-test for independent samples. Sample size calculation was done using G Power and Sample Size Calculations software, version 3.0.11 for MS Windows (William D. Dupont and Walton D., Vanderbilt University, Nashville, Tennessee, USA).

The study was registered in www.clinicaltrials.gov with the linkhttps://register.clinicaltrials.gov/prs/app/action/SelectProtocol?sid=S000BVYX&selectaction=Edit&uid=U00065KS&ts=17&cx=t01ek8. The study has been approved by Research Ethics Committee in Faculty of Dentistry, Cairo University with number 6/2/22. The study was conducted by the Helsinki Declaration which was revised in 2013 [21].

The main outcomes of the study are the expression of miRNA-93 and miRNA-412-3p in OLP, OSCC, and controls. Secondary outcomes include the diagnostic accuracy of miRNA-93 and miRNA-412-3p for OLP and OSCC and the relation between both criteria and other variables like age, gender, OLP form, OLP lesion size, and severity of symptoms, tumor size, tumor grade, and pain score. The only parameters compared between all groups are age and gender, but still, there were specific parameters for group I as OLP size of lesion, OLP intensity, and pain score, and for group III was tumor grade.

To assess the clinical symptoms, the criteria established by Thongprasom et al. were utilized [22]. These criteria allowed for the evaluation of the severity of lesions and symptoms in patients diagnosed with oral lichen planus. The intensity of the symptoms was assessed according to Lodi et al*.* [23]. Pain score was measured according to the Numerical Rating Scale (NRS) [24].

The salivary samples were obtained for Group I, II, and III patients. Unstimulated salivary samples were collected from all participants utilizing standard techniques described by Navazesh [25]. Before salivary sample collection by ½ an hour the subjects were asked to withhold eating, smoking, or drinking. Collecting samples was performed in the morning by having the participants incline their heads forward to deliver saliva in a sterile test tube after swallowing. All samples were given codes and serial numbers before being sent for evaluation in the biochemistry lab. Each saliva sample was collected in a Falcon tube (50 ml size) on ice. Approximately 400 µl (The minimum RNA concentration to proceed for PCR was 60ug) of unstimulated whole saliva was collected between 5–10 min and then stored at -80 °C till PCR assay. Each participant’s oral cavity will be rinsed thoroughly with water and saliva, and the resulting mixture will be carefully collected in a Falcon tube with a capacity of 50 ml. This collection process will be carried out while maintaining a low temperature by placing the Falcon tube on ice. To obtain unstimulated whole saliva, approximately 400 μl will be collected within a time frame of 5 to 10 minutes [26]. Each participant was provided an ID NO. for data management and labeling of the samples. Just after saliva collection, the specimen was snap-frozen in liquid nitrogen or packed in ice.

### Detection and assessment of salivary miRNA-93 and miRNA-412-3p level

#### MicroRNA extraction from saliva

The extraction of micro-RNAs from saliva was carried out using the miRNA easy extraction kit (Qiagen, Valencia, CA, USA). The extraction process involved the use of 1000 μL QIAzol lysis reagent to extract from 200 μL saliva. After homogenization, the homogenate was incubated for 5 min at RT. Then, 200 μL chloroform was added, vortexed for 15 sec, and incubated for 2–3 min at ambient temperature. This was followed by centrifugation at 12,000 × *g* at 4 °C for 15 min. After removing the upper watery phase, 1.5 times the volume of ethanol (100%) was added. Then, 700 uL of this mixture was transferred to an RNA easy Mini spin column placed in a 2 ml collection tube. The centrifugation was carried out at room temperature for 15 s at a speed of 8000 × *g*. Once the mixture had passed through the column, 700 μL of buffer RWT was introduced to each column and centrifuged at 8000 × *g* for 15 s at room temperature. Following this, 500 μL of buffer RPE was added to the column and centrifuged at 8000 × *g* for 15 s at room temperature. This process was repeated for 2 minutes at full speed. The column was then moved to a new 1.5 ml collection tube and 50 uL of RNase-free water was pipetted onto the column and centrifuged for 1 minute at 8000 xg to elute RNA. Finally, the extracted micro-RNA was stored at –80 °C until use [27].

#### Reverse transcription (RT) and real-time quantitative PCR (qPCR)

Reverse transcription was carried out on microRNA that was extracted from saliva in a final volume of 20 uL RT reactions (incubated for 60 min at 37 °C, followed by 5 min at 95 °C) using the miScript II RT kit (Qiagen, Valencia, CA, USA) according to the manufacturer’s instructions. Real-time qPCR was conducted utilizing the MiScript SYBR Green PCR kit from Qiagen, located in Valencia, CA, USA. The miScript primer assay miRNA-93 and miRNA-412-3p from the same company were employed in this experiment. A total volume of 20 μL reaction was prepared, with 20 ng of cDNA serving as the template. The experiment was carried out under the specified conditions: denaturation at 95 °C for 15 min followed by 40 cycles of 94 °C for 15 s, 55 °C for 30 s, and 70 °C for 30 s, in which fluorescence was acquired and detected by Rotor-gene Q Real-time PCR system (Qiagen, USA). After the PCR cycles, melting curve analyses were performed to validate the specific generation of the expected PCR product. SNORD was used as an endogenous control. The expression level was evaluated using the ΔCt method. The cycle threshold (Ct) value is the number of qPCR cycles compulsory for the fluorescent signal to cross a specified threshold. ΔCt was calculated by subtracting the Ct values of SNORD from those of target micro-RNAs. The ΔΔCt was derived by subtracting the ΔCt value of the control samples from the ΔCt value of the cancer samples. The alteration in magnitude, known as the fold change, in miR-21 expression was calculated by the equation 2–ΔΔCt [28].

### Statistical methods

Categorical data were presented as frequencies and percentages and were analyzed using the chi-square test. Numerical data were tested for normality using the Shapiro-Wilk test. Normally distributed data were presented as mean and standard deviation values and were compared using one-way ANOVA followed by Tukey’s post hoc test. Pain scores are only measured in a single group (Group I), so intergroup comparisons cannot be made (i.e., there are no scores in other groups to compare with). A correlation coefficient was used to correlate pain scores measured in the cases of group I with their respective markers’ levels. Descriptive statistics (mean, SD median, etc.) were used to describe the measured score in this group. Correlations were analyzed using Spearman’s rank-order correlation coefficient. ROC curves were constructed to determine the diagnostic accuracy of different markers and were tested for statistical significance using a z-test. Linear regression models were built to assess the effect of different studied predictors on markers’ levels. The normality and variance homogeneity assumptions were evaluated by viewing the distribution and using Shapiro-Wilk’s and Levene’s tests, respectively. The significance level was set at *p* < 0.05 within all tests. Statistical analysis was performed with R statistical analysis software version 4.3.1 for Windows.

## Results

The study was applied to 60 patients with a range of ages from 20 to 70 years old. In Group I female patients were more than males, unlike in Group III. Both genders are equal in number. The demographic data of each group is described in Table 1. Descriptive statistics for Group I and Group III are presented in Tables 2 and 3.

**Table 1:** Intergroup comparison and summary statistics for demographic data.

Parameter		Group I	Group II	Group III	*p* value
**Sex [n (%)]**	**Male**	9 (45.0%)	10 (50.0%)	15 (75.0%)	**0.122** **ns**
	**Female**	11 (55.0%)	10 (50.0%)	5 (25.0%)	
**Age (Mean** **±** **SD)**		45.70 ± 12.33^B^	40.25 ± 11.67^B^	55.85 ± 12.42^A^	<0.001*

Superscripts "A" and "B" and the asterisk "*" indicate statistical significance and grouping.

**Table 2:** Descriptive statistics for Group I.

Parameter		Value
Type of OLP [*n* (%)]	Reticular	7 (35.0%)
	Atrophic	4 (20.0%)
	Bullous-Erosive	9 (45.0%)
Lesion size [*n* (%)]	Mild white striae without erythematous or erosive	2 (10.0%)
	White striae with atrophic area < 1 cm^2^	3 (15.0%)
	White striae with atrophic area > 1 cm^2^	4 (20.0%)
	White striae with erosive area < 1 cm^2^	3 (15.0%)
	White striae with erosive area > 1 cm^2^	8 (40.0%)
Lesion severity [*n* (%)]	Mild	6 (30.0%)
	Moderate	6 (30.0%)
	Severe	8 (40.0%)
Pain score	Mean ± SD	6.80 ± 2.82
	Median (IQR)	7.00 (5.50)

**Table 3:** Descriptive statistics for Group III.

Parameter		Value
**Site of tumor [*n*** **(%)]**	**Maxilla**	1 (5.0%)
	**Hard palate**	5 (25.0%)
	**Mandible**	3 (15.0%)
	**Tongue**	11 (55.0%)
**Tumor grade [*n*** **(%)]**	**(I)**	1 (5.0%)
	**(II)**	9 (45.0%)
	**(III)**	10 (50.0%)

**Fig. 3:**
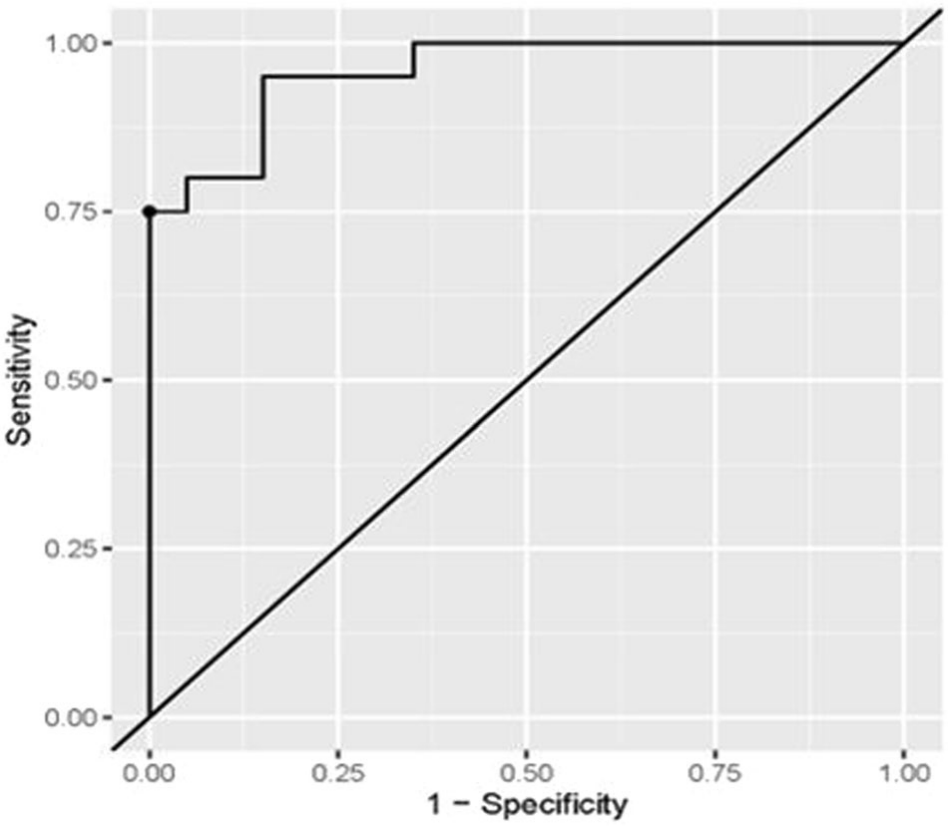
ROC curve for the accuracy of miRNA-93 in diagnosis of OLP in Group I.

**Fig. 4:**
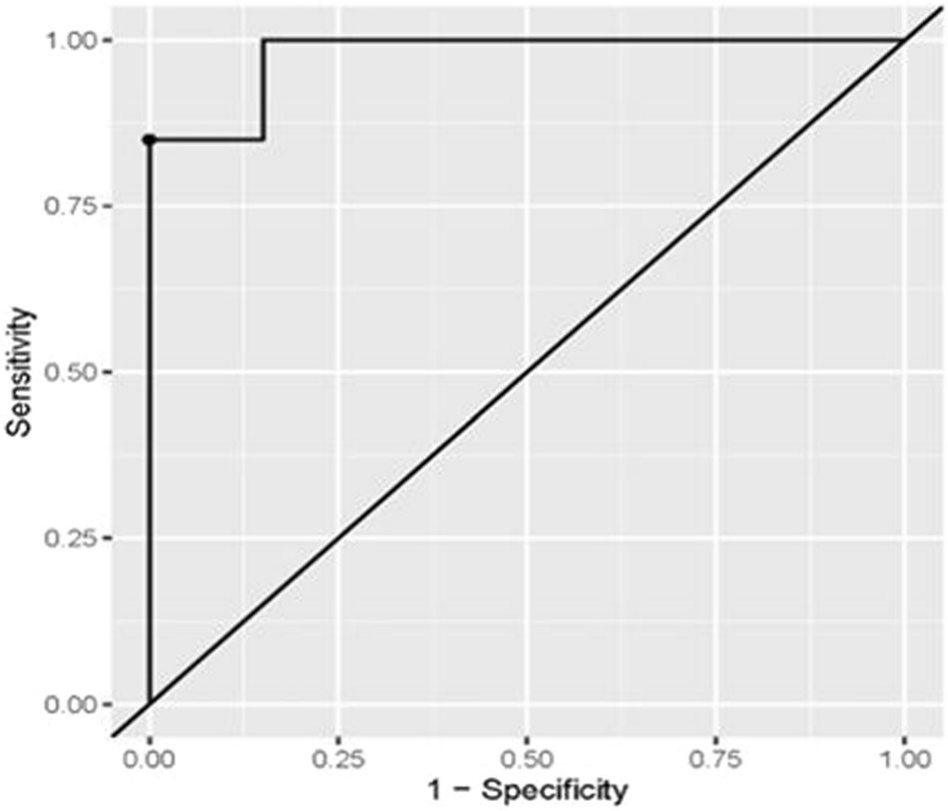
ROC curve for the accuracy of miR-93 in diagnosis of OSCC in Group III.

Both miRNA-93 and miRNA412-3p were more highly expressed in Group III than in Group I control, with a statistically significant difference. MiRNA412-3p has shown higher expression than miRNA-93 in all groups (Figs. 3 and  4).

**Fig. 5:**
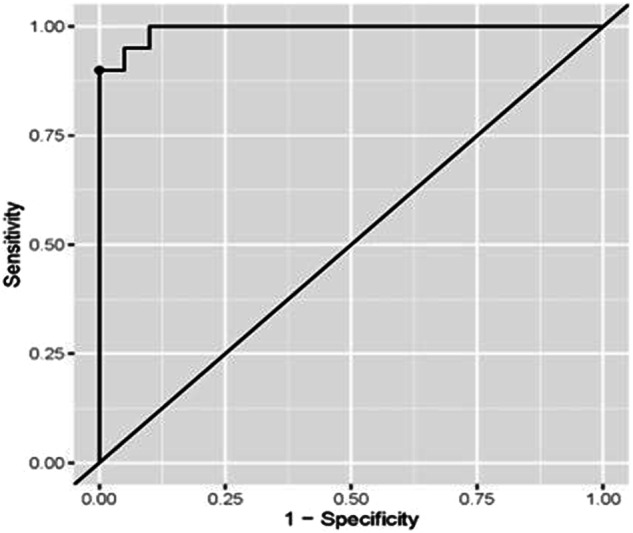
ROC curve for the accuracy of miR-412-3p in diagnosis of OLP in Group I.

ROC curve analysis for the diagnostic accuracy of miRNA-93 in the diagnosis of LP was presented in Table 4 and Fig. 5 The optimal cut-off (>=1.44) value was determined based on the Youden index. The associated sensitivity and specificity were (95%) indicating a high probability of higher miR-93 levels when the disease is present and lower levels when the disease is not present. The (PPV) was (94.74%) indicating a high probability of the disease’s presence when the levels of miR-93 levels are high. The (NPV) was (94.12%) indicating a high probability of the disease’s absence when the levels of the marker are low. (AUC) was (0.958) and was significantly different from (0.5) (*p* < 0.001), indicating the higher ability of the marker to distinguish between healthy and diseased subjects.

**Table 4:** Coordinates of ROC curve analysis for diagnosis of OLP in Group I using miRNA-93.

Parameter	Value [95%CI]
**Sensitivity**	95.00% (75.00%:100.00%)
**Specificity**	95.00% (70.00%:100.00%)
**Cut off point**	>=1.44
**Positive predictive value (PPV)**	94.74% (80.00%:100.00%)
**Negative predictive value (NPV)**	94.12% (76.92%:100.00%)
**Area under the curve (AUC)**	0.958 (0.905:1.000)

**Fig. 6:**
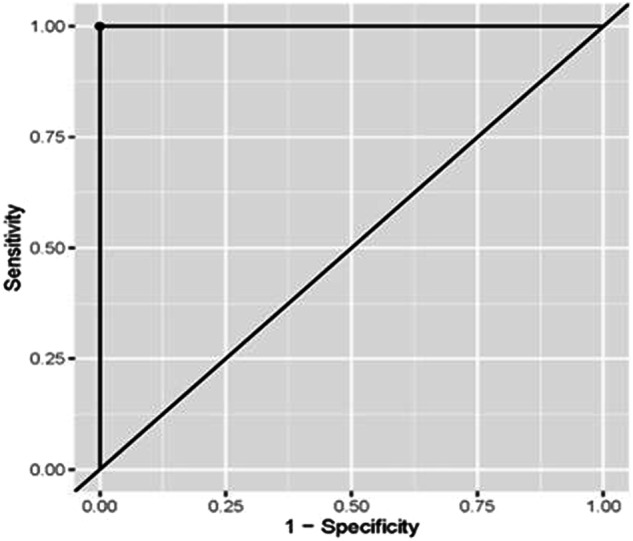
ROC curve for the accuracy of miR-412-3p in diagnosis of OSCC in Group III.

**Fig. 7:**
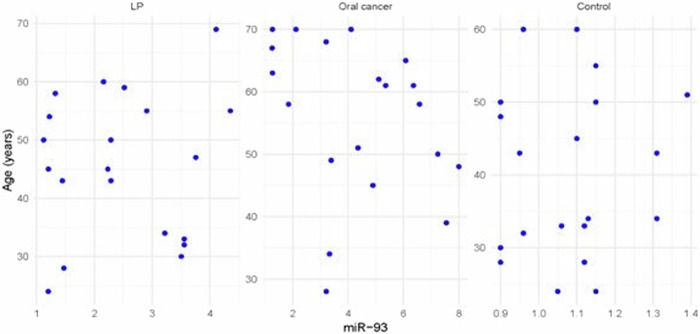
Scatter plot showing the correlation between miRNA-93 and age in all groups.

**Fig. 8:**
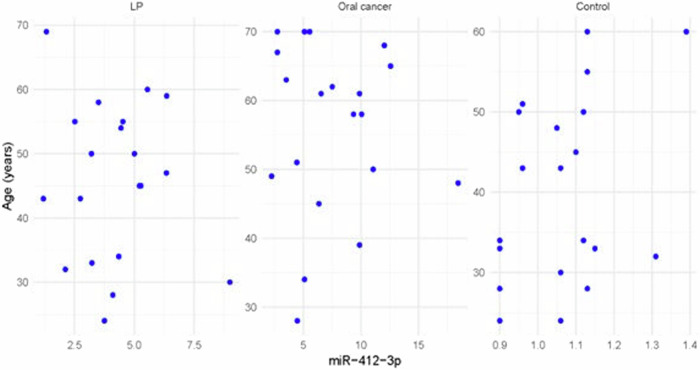
Scatter plot showing the correlation between miR-412-3p and age in all groups.

ROC curve analyses were conducted to evaluate the diagnostic accuracy of miRNA-93 for detecting oral cancer and miRNA-412-3p for detecting both OLP and oral cancer. The associated sensitivity and specificity values were (100%), suggesting that these markers accurately identify the presence of the disease when elevated and correctly indicate its absence when levels are lower. The positive predictive value (PPV) and negative predictive value (NPV) were also (100%), reflecting a high likelihood that the disease is present when marker levels are elevated and absent when marker levels are lower. The optimal cut-off values were determined as (≥1.49), (≥1.48), and (≥1.50), respectively, based on the Youden index criteria. The area under the curve (AUC) values were (0.978), (0.992), and [1], respectively, demonstrating the strong discriminative ability of these markers in distinguishing between healthy and diseased individuals. These findings are detailed in Tables 5–7 and Figs. 6–8.

**Table 5:** Coordinates of ROC curve analysis for diagnosis of OSCC in Group III using miRNA-93.

Parameter	Value [95%CI]
**Sensitivity**	100.00% (80.00%:100.00%)
**Specificity**	100.00% (80.00%:100.00%)
**Cut off point**	>=1.49
**Positive predictive value (PPV)**	100.00% (83.33%:100.00%)
**Negative predictive value (NPV)**	100.00% (83.33%:100.00%)
**Area under the curve (AUC)**	0.978 (0.943:1.000)

**Table 6:** Coordinates of ROC curve analysis for diagnosis of OLP in Group I using miRNA-412-3p.

Parameter	Value [95%CI]
**Sensitivity**	100.00% (85.00%:100.00%)
**Specificity**	100.00% (85.00%:100.00%)
**Cut off point**	>=1.48
**Positive predictive value (PPV)**	100.00% (86.96%:100.00%)
**Negative predictive value (NPV)**	100.00% (86.96%:100.00%)
**Area under the curve (AUC)**	0.992 (0.977:1.000)

**Table 7:** Coordinates of ROC curve analysis for diagnosis of OSCC in Group III using miRNA-412-3p.

Parameter	Value [95%CI]
**Sensitivity**	100.00% (100.00%:100.00%)
**Specificity**	100.00% (100.00%:100.00%)
**Cut off point**	>=1.50
**Positive predictive value (PPV)**	100.00% (100.00%:100.00%)
**Negative predictive value (NPV)**	100.00% (100.00%:100.00%)
**Area under the curve (AUC)**	1.000 (1.000:1.000)

**Fig. 9:**
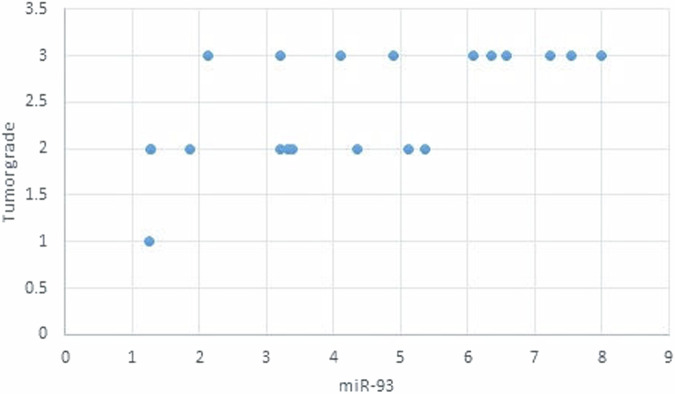
Scatter plot showing the correlation between miRNA-93 and tumor grade in Group III.

**Fig. 10:**
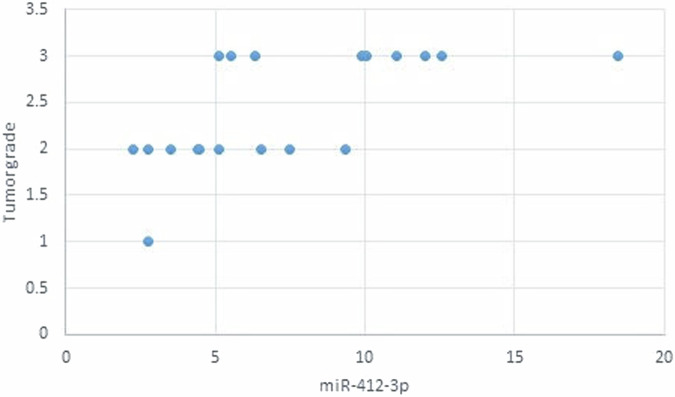
Scatter plot showing the correlation between miRNA-412-3p and tumor grade in Group III.

**Fig. 11:**
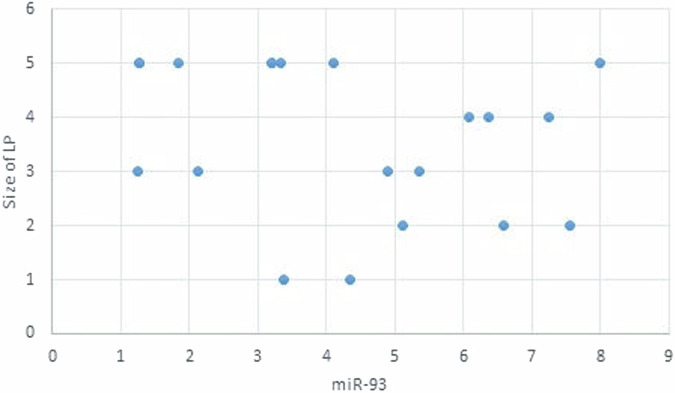
Scatter plot showing the correlation between miRNA-93 and lesion size in Group I.

**Fig. 12:**
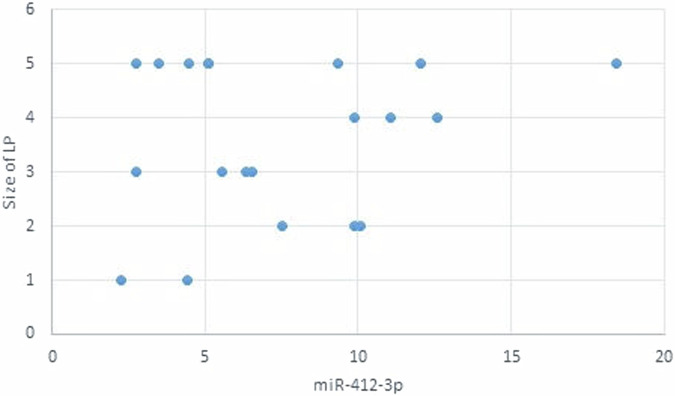
Scatter plot showing the correlation between miRNA-412-3p and lesion size in Group I.

**Fig. 13:**
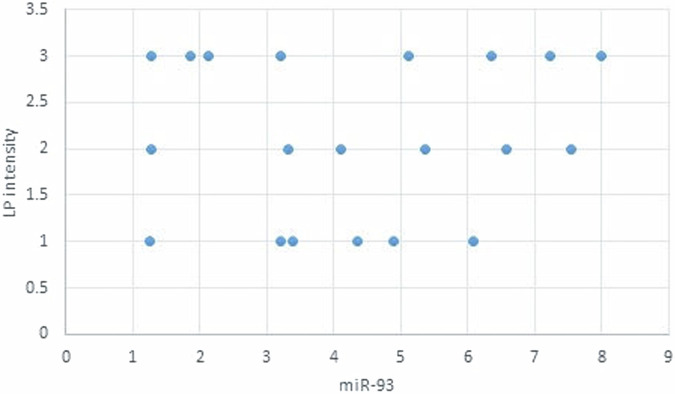
Scatter plot showing the correlation between miRNA-93 and OLP intensity in Group I.

**Fig. 14:**
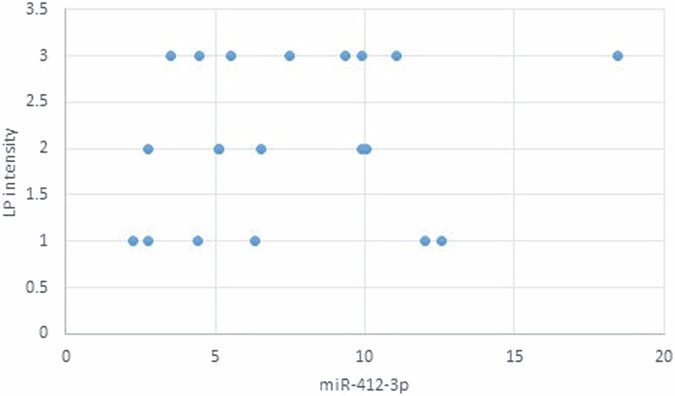
Scatter plot showing the correlation between miRNA-412-3p and OLP intensity in Group I.

**Fig. 15:**
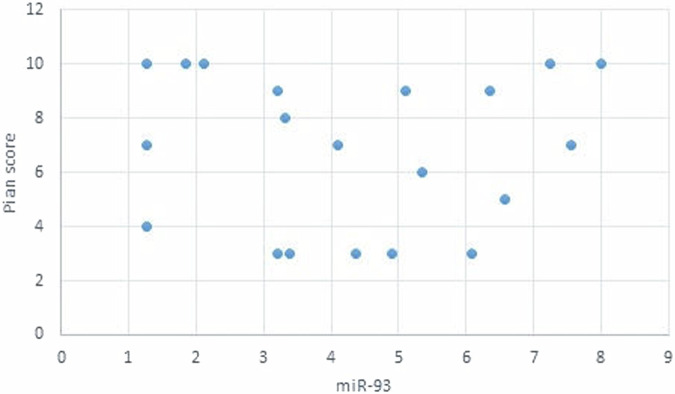
Scatter plot showing the correlation between miRNA-93 and Pain score in Group I.

The statistical analysis has shown no correlation between miRNA-93 and miRNA-412-3p and the age or gender of patients in all groups and the size of OLP lesions in Group I. MiRNA–93 has no correlation with OLP type, OLP intensity, and pain score in Group I unlike miRNA-412-3p. Both markers have a strong positive correlation with tumor grade. These results are demonstrated in Tables 7–21 and Figs. 9–15.

**Table 8:** Correlation between miRNA-93 and gender.

Group	miRNA-93 (Mean ± SD)		*p* value
	Male	Female	
**GROUP I**	2.49 ± 0.92	2.45 ± 1.24	**0.935** **ns**
**Group II**	1.05 ± 0.18	1.11 ± 0.10	**0.314** **ns**
**Group III**	3.83 ± 2.22	5.82 ± 1.23	**0.075** **ns**

**Table 9:** Correlation between miRNA-412-3p and gender.

Group	miRNA-412-3p (Mean ± SD)		*p* value
	Male	Female	
**GROUP I**	4.56 ± 2.31	3.89 ± 1.47	**0.441** **ns**
**Group II**	1.10 ± 0.15	1.03 ± 0.12	**0.280** **ns**
**Group III**	6.88 ± 4.38	9.26 ± 2.97	**0.277** **ns**

**Table 10:** Correlation between miRNA-93 and age.

Group	Correlation coefficient (95% CI)	*p* value
**GROUP I**	**0.099 (−0.360:0.519)**	**0.679** **ns**
**Group II**	**0.141 (−0.321:0.549)**	**0.553** **ns**
**Group III**	**−0.430 (−0.733:0.016)**	**0.059** **ns**

**Table 11:** Correlation between miRNA-412-3p and age.

Group	Correlation coefficient (95% CI)	*p* value
**GROUP I**	**0.086 (−0.371:0.509)**	**0.719** **ns**
**Group III**	**−0.095 (−0.516:0.363)**	**0.691** **ns**
**Group II**	**0.294 (−0.171:0.651)**	**0.209** **ns**

**Table 12:** Association between miRNA-93 and OLP type in Group I.

miR-93 (Mean ± SD)			*p* value
Reticular	Atrophic	Bullous-Erosive	
2.37 ± 1.19 ^A^	2.10 ± 1.09 ^A^	2.71 ± 1.06 ^**A**^	**0.639** **ns**

Superscript A indicates that the mean values for miR-93 in the Reticular, Atrophic, and Bullous-Erosive groups are statistically similar.

**Table 13:** Association between miRNA-412-3p and OLP type in Group I.

miR-412-3p (Mean ± SD)			*p* value
Reticular	Atrophic	Bullous-Erosive	
3.19 ± 1.34^B^	3.05 ± 1.57^B^	5.48 ± 1.62 ^A^	0.012*

The superscripts "A" and "B" indicate the results of post hoc comparisons. Superscript A: the Bullous-Erosive group is significantly different from the Reticular and Atrophic groups. Superscript B: the Reticular and Atrophic groups are not significantly different from each other, but both are different from the Bullous-Erosive group. The asterisk next to the p value (0.012*) denotes statistical significance.

**Table 14:** Correlation between miRNA-93 and tumor grade in Group III.

Correlation coefficient (95% CI)	*p* value
**0.613 (0.233:0.830)**	0.004*

Asterisk (*) indicates that the *p* value is statistically significant.

**Table 15:** Correlation between miRNA-412-3p and tumor grade in Group III.

Correlation coefficient (95% CI)	*p* value
**0.721 (0.410:0.882)**	<0.001*

Asterisk (*) indicates that the *p* value is statistically significant.

**Table 16:** Correlation between miRNA-93 and lesion size in Group I.

Correlation coefficient (95% CI)	*p* value
**0.070 (−0.385:0.497)**	**0.770** **ns**

**Table 17:** Correlation between miRNA-412-3p and lesion size in Group I.

Correlation coefficient (95% CI)	*p* value
**0.296 (−0.169:0.653)**	**0.205** **ns**

**Table 18:** Correlation between miRNA-93 and lesion intensity in Group I.

Correlation coefficient (95% CI)	*p* value
**0.178 (−0.288:0.575)**	**0.454** **ns**

**Table 19:** Correlation between miRNA-412-3p and lesion intensity in Group I.

Correlation coefficient (95% CI)	*p* value
**0.614 (0.236:0.831)**	0.004*

Asterisk (*) indicates that the *p* value is statistically significant.

**Table 20:** Correlation between miRNA-93 and pain score in Group I.

Correlation coefficient (95% CI)	*p* value
**0.130 (−0.331:0.542)**	**0.584** **ns**

**Table 21:** Correlation between miRNA-93 and pain score in Group I.

Correlation coefficient (95% CI)	*p* value
**0.558 (0.153:0.802)**	0.011*

Asterisk (*) indicates that the *p* value is statistically significant.

**Fig. 16:**
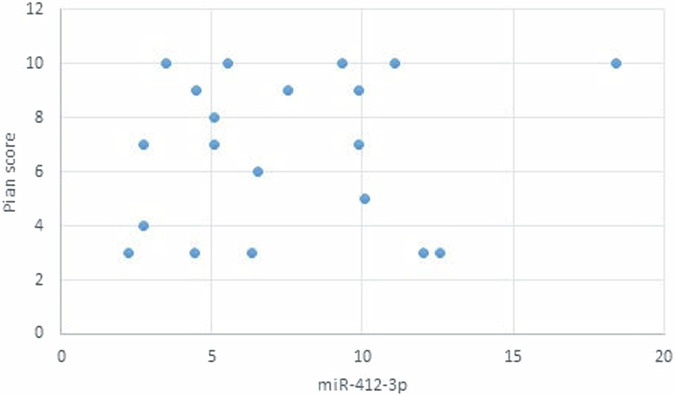
Scatter plot showing the correlation between miRNA-412-3p and Pain score in Group I.

The assumptions were valid for all models. The original predictors for group I were (age, gender, intensity of symptoms, pain score, and lesion size). Due to high Variance Inflation Factor (VIF) values (57.76 for intensity of symptoms and 36.05 for pain score), new models were constructed with the intensity of symptoms predictor removed. The revised models showed all VIF values below 3 as shown in Fig. 16.

For group I, both miRNA-93 (*f* = 0.49, *p* = 0.826) and miRNA-412-3p (*f* = 1.09, *p* = 0.428) models were not statistically significant.

For group III, the miRNA-93 model was statistically significant (*f* = 5.97, *p* = 0.004) and predicted 51.1% (adjusted R2) of the variability in the marker level. Out of the studied predictors (i.e., age, sex, and tumor grade), only age significantly contributed to the model (t = −2.56, *p* = 0.022), with younger age being associated with increased marker level. The miRNA-412-3p model was not statistically significant (*f* = 3.04, *p* = 0.051) (Tables 22 and 23).

**Table 22:** Regression models for the Group I.

Marker	Parameter	Coefficient	Confidence level		SE	*t* value	*p* value
			Lower	Upper			
*miR-93*	***(Intercept)***	1.36	−2.29	5.00	1.67	0.81	**0.433**
	***Age***	0.03	−0.04	0.09	0.03	0.92	**0.378**
	***Sex (Female)***	−0.11	−1.31	1.08	0.55	−0.21	**0.840**
	***Pain score***	0.17	−0.12	0.46	0.13	1.29	**0.220**
	***Lesion size (White striae with atrophic area*** < ***1*** ***cm***^***2***^***)***	−1.55	−4.62	1.51	1.41	−1.11	**0.290**
	***Lesion size (White striae with atrophic area*** > ***1*** ***cm***^***2***^***)***	−1.60	−4.01	0.81	1.11	−1.44	**0.174**
	***Lesion size (white striae with erosive area*** < ***1*** ***cm***^***2***^***)***	−0.93	−3.59	1.73	1.22	−0.76	**0.463**
	***Lesion size (white striae with erosive area*** > ***1*** ***cm***^***2***^***)***	−1.32	−3.97	1.33	1.22	−1.09	**0.298**
*miR-412-3p*	***(Intercept)***	1.02	−4.56	6.60	2.56	0.40	**0.697**
	***Age***	0.02	−0.08	0.12	0.05	0.48	**0.636**
	***Sex (Female)***	−0.86	−2.69	0.97	0.84	−1.03	**0.325**
	***Pain score***	0.39	−0.05	0.83	0.20	1.94	**0.076**
	***Lesion size (White striae with atrophic area*** < ***1*** ***cm***^***2***^***)***	−0.44	−5.13	4.25	2.15	−0.20	**0.842**
	***Lesion size (White striae with atrophic area*** > ***1*** ***cm***^***2***^***)***	−0.04	−3.74	3.65	1.70	−0.02	**0.981**
	***Lesion size (white striae with erosive area*** < ***1*** ***cm***^***2***^***)***	1.05	−3.02	5.12	1.87	0.56	**0.584**
	***Lesion size (white striae with erosive area*** > ***1*** ***cm***^***2***^***)***	−0.29	−4.34	3.77	1.86	−0.15	**0.880**

**Table 23:** Regression models for the group III.

*Marker*	*Parameter*	*Coefficient*	*Confidence level*		*SE*	*t* value	*p* value
			*Lower*	*Upper*			
*miR-93*	***(Intercept)***	6.43	1.03	11.82	2.53	2.54	**0.023**^**a**^
	***Age***	−0.08	−0.14	−0.01	0.03	−2.56	**0.022**^**a**^
	***Sex (Female)***	1.57	−0.27	3.41	0.86	1.81	**0.090**
	***Grade (II)***	0.72	−2.84	4.28	1.67	0.43	**0.673**
	***Grade (III)***	2.99	−0.58	6.56	1.67	1.79	**0.094**
*miR-412-3p*	***(Intercept)***	5.26	−6.99	17.51	5.75	0.92	**0.374**
	***Age***	−0.04	−0.18	0.11	0.07	−0.55	**0.592**
	***Sex (Female)***	0.51	−3.67	4.70	1.96	0.26	**0.798**
	***Grade (II)***	1.77	−6.31	9.85	3.79	0.47	**0.647**
	***Grade (III)***	6.78	−1.32	14.88	3.80	1.78	**0.095**

^a^Significant (*p* < 0.05).

## Discussion

Oral Lichen Planus is one of the most common mucocutaneous diseases. It is an immune condition mainly caused by immune changes in the affected tissues. It may affect other non-oral tissues such as skin or genitalia. Its prevalence shows some female predilection. It has multiple forms may be non-erosive forms or erosive forms with more malignant transformation potential and aggressive symptoms than the other forms. OLP is classified as a potentially malignant disease as it has a rare malignant transformation potential reaching 1%. So, OLP patients must be well diagnosed, managed, and followed up every 3 months [29].

This study estimated the salivary level of microRNA-93 and microRNA-412-3p biomarkers in patients with OLP and OSCC. The results showed that the expression of microRNA-93 and microRNA-412-3p was upregulated in OSCC than OLP than the controls in a statistically significant difference for all intergroup comparisons (*P* < 0.001). Expression of both microRNAs had no significant correlation with age or gender.

Over-expression of both microRNA-93 and microRNA-412-3p -93 in OLP patients indicates the involvement of these markers in the pathogenesis of OLP. Also, altered expression of this biomarker has been reported in OSCC [30–32]. Gai et al*.* have diagnosed the over-expression of microRNA-412-3p in OSCC. The results were statistically significant and the AUC was 0.871. these results are approximately similar to this study [17]

Alteration of expression of microRNAs in the serum, blood, plasma, and saliva samples of patients with head and neck cancer had gained great concern as potential biomarkers for early detection or determination of prognosis [33]. Other studies have discussed the role of microRNAs in the pathogenesis and severity of OLP [34, 35]. However, tissue and serum samples of OLP patients were mainly assessed [36].

Other microRNAs were examined in OLP and OSCC. Liang et al. estimated the expression of microRNA-155, microRNA-146a, and microRNA-146b in peripheral blood mononuclear cells and tissue samples of OLP patients and healthy controls [35]. Moreover, they analyzed the correlation of microRNA expression and clinical features of OLP. The results of RT-PCR showed that the expression of microRNA-155, and microRNA-146a in peripheral blood mononuclear cells of OLP patients was significantly higher than that in controls, which was in line with the present results. However, in contrast to the present findings, they found no significant difference in the expression of microRNA-146a in peripheral blood mononuclear cells between the OLP and control groups. These results come in line with a recent study by Mehdipour et al. [37].

Additionally, Mehdipour et al. have diagnosed the over-expression of salivary expression of miR-21 and miR-31 in OLP and OSCC than controls and a rise in expression of miR-125a and miR-200a in controls than both OLP and OSCC. MiR-21 and miR-31 may have a role in inflammatory processes in OLP and malignant transformation in OSCC. The decrease of miR-125a and miR-200a should confirm that these markers can be considered to detect the prognosis of OSCC. The results of miR-21 and miR-125a were statistically significant, unlike the other markers in the study [38].

Liu et al. evaluated the expression of microRNA-146a in peripheral blood CD4 + T-cells and local OLP lesions, and its association with clinical presentation of OLP using RT-PCR. They found no significant difference in the expression of microRNA-146a by the peripheral blood CD4 + T-cells between the OLP and control groups, which was different from the present findings. This difference may be because they analyzed serum samples while this study assessed the saliva samples [39].

The limitation of this study is the limited number of patients and just the systemically healthy OLP patients. The limited number is due to the cost factor and a limited number of sampling kits for the study as we compare two miRNAs within 3 groups. Our recommendations are to broaden the diseased groups to include systemically diseased OLP and to compare OLP patients with various systemic diseases. We suggest follow-up for OLP cases to compare the concentration of markers before and after disease management. Confirmation of the obtained results with a larger sample size is recommended. Finally, we recommend extending studies to include more suspected micro RNAs as potential biomarkers in the pathogenesis of OLP and as oncogenic biomarkers.

## Conclusion

Both miRNA-93 and miRNA-412-3p can be used as biomarkers for diagnosis and prediction of the oncogenic potential of OLP.

## Data Availability

The original contributions presented in the study are included in the article, further inquiries can be directed to the corresponding author.
